# Debugging Eukaryotic Genetic Code Expansion for Site‐Specific Click‐PAINT Super‐Resolution Microscopy

**DOI:** 10.1002/anie.201608284

**Published:** 2016-11-02

**Authors:** Ivana Nikić, Gemma Estrada Girona, Jun Hee Kang, Giulia Paci, Sofya Mikhaleva, Christine Koehler, Nataliia V. Shymanska, Camilla Ventura Santos, Daniel Spitz, Edward A. Lemke

**Affiliations:** ^1^ Structural and Computational Biology Unit, Cell Biology and Biophysics UnitEMBLMeyerhofstrasse 169117HeidelbergGermany; ^2^ Present address: Werner Reichardt Centre for Integrative, NeuroscienceUniversity of TübingenTübingen72076Germany

**Keywords:** click chemistry, genetic code expansion, PAINT, protein labeling, super-resolution microscopy

## Abstract

Super‐resolution microscopy (SRM) greatly benefits from the ability to install small photostable fluorescent labels into proteins. Genetic code expansion (GCE) technology addresses this demand, allowing the introduction of small labeling sites, in the form of uniquely reactive noncanonical amino acids (ncAAs), at any residue in a target protein. However, low incorporation efficiency of ncAAs and high background fluorescence limit its current SRM applications. Redirecting the subcellular localization of the pyrrolysine‐based GCE system for click chemistry, combined with DNA‐PAINT microscopy, enables the visualization of even low‐abundance proteins inside mammalian cells. This approach links a versatile, biocompatible, and potentially unbleachable labeling method with residue‐specific precision. Moreover, our reengineered GCE system eliminates untargeted background fluorescence and substantially boosts the expression yield, which is of general interest for enhanced protein engineering in eukaryotes using GCE.

Fluorescence microscopy in general and super‐resolution microscopy (SRM) in particular can benefit from the use of small and photostable fluorophores. The challenge of direct and specific protein labeling with organic fluorophores inside mammalian cells can be addressed through the genetic encoding of dye coupling modules, such as various protein or peptide tags (for a more comprehensive overview of powerful technologies see Ref. 1). One of the most versatile methods to achieve labeling in a residue specific fashion is the incorporation of noncanonical amino acids (ncAAs) into proteins by using genetic code expansion (GCE). GCE most commonly relies on Amber (TAG) stop codon suppression by means of a tRNA and aminoacyl tRNA synthetase (tRNA/RS) pair orthogonal to the host translational machinery. The RS is typically engineered in such a way that it only accepts the ncAA of choice, which can simply be added to the growth medium. This leads to acylation of the cognate tRNA^CUA^ only when the ncAA is present, thereby resulting in its residue‐specific incorporation in response to an artificially introduced UAG codon in the mRNA coding for the protein of interest (POI, for reviews, see Ref. 3).

We and others have recently shown that ncAAs containing strained alkyne or alkene moieties can be encoded in living mammalian cells by means of the pyrrolysine tRNA^Pyl^/PylRS pair from *Methanosarcina*.^4^ Cyclooctyne and *trans*‐cyclooctene amino acid derivatives can subsequently be labeled through click chemistry reactions, such as ultrafast and bioorthogonal strain‐promoted inverse‐electron‐demand Diels–Alder cycloadditions (SPIEDAC) with 1,2,4,5‐tetrazines. Even though such reactions have previously been used to label and study surface proteins and highly abundant cytoskeletal proteins in mammalian cells with SRM,^5^ applications to less abundant proteins are largely obscured by the limited efficiency of the GCE system, nonspecific binding (sticking) of the dyes, as well as frequent and highly fluorescent background in the nucleus, particularly in the nucleolus.4d, 5b This renders an entire major organelle almost inaccessible to SRM through GCE‐based labeling.

To improve the potential of GCE for SRM applications, we first aimed to understand the origin of and eliminate the nonspecific nuclear background labeling. We analyzed the widely used *M. mazei* PylRS protein sequence and, to our surprise, identified a putative nuclear localization sequence (NLS; Figure S1 in the Supporting Information). NLSs are small motifs that direct proteins to the nuclear import machinery, which relocates NLS‐bearing proteins into the nucleus.^6^ This finding is indeed unexpected, given that archaea, the domain that *Methanosarcina* belong to, do not possess a nucleus. To test whether PylRS is indeed localized to the nucleus, we first recombinantly expressed PylRS from *M. mazei* in *E. coli* to generate a polyclonal antibody (Ab_PylRS_; Figure S2 in the Supporting Information). Immunofluorescence (IF) staining with Ab_PylRS_ of HEK293T and COS‐7 cells (HEK and COS) expressing the tRNA^Pyl^/PylRS^AF^ (AF refers to a previously described PylRS mutant that accepts bulky side‐chain moieties such as *t*‐butyloxycarbonyl (BOC)‐ and *trans*‐cyclooctene ncAAs)4a,4d, 5a, 7 system revealed clear localization of the PylRS to the nucleus (see Figure 1 for HEK cells and Figure S3 for COS cells). Since PylRS has a high affinity for its cognate tRNA^Pyl^, we used fluorescence in situ hybridization (FISH; see Figure 1 c and Figure S3 for COS) to confirm that tRNA^Pyl^ is also mainly localized to the nucleus.

**Figure 1:**
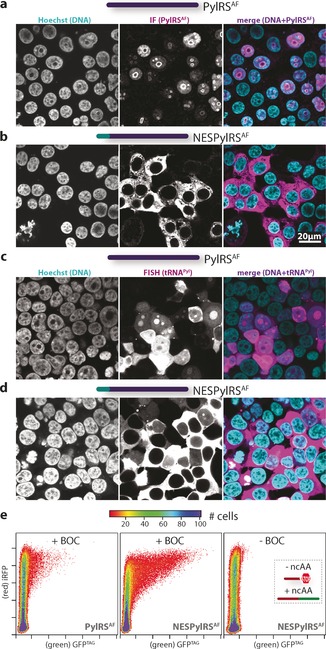
a,b) Immunofluorescence staining of HEK cells expressing either tRNA^Pyl^/PylRS^AF^ (a) or tRNA^Pyl^/NESPylRS^AF^ (b). Left panel: Hoechst 33342, central panel: Ab_PylRS_ staining, right panel: merge. For tRNA^Pyl^/NESPylRS^AF^, only cytoplasmic staining is visible (see Figure S3 for COS cells). c,d) Fluorescence in situ hybridization of tRNA^Pyl^ in HEK cells expressing either tRNA^Pyl^/PylRS^AF^ (c) or tRNA^Pyl^/NESPylRS^AF^ (d). Left panel: Hoechst 33342, central panel: anti‐DIG‐fluorescein channel (tRNA), right panel: merge. For tRNA^Pyl^/NESPylRS^AF^, cytoplasmic staining is clearly visible, in contrast to a much more heterogeneous and strong nucleolar signal for the tRNA^Pyl^/PylRS^AF^‐transfected cells (see Figure S3 for COS cells). e) Flow cytometry analysis of the reporter iRFP–GFP^Y39TAG^ to assess the Amber suppression efficiency in the presence of BOC of PylRS^AF^ (left) and NESPylRS^AF^ (center), and of NESPylRS^AF^ without ncAA (right). The analysis shows that the number of bright GFP‐expressing cells (i.e. successful Amber suppression) is substantially enhanced for the NESPylRS^AF^ in the presence of BOC (up to 15‐fold, shown here is the average of a full titration, which is detailed in Figure S4). The axes indicate fluorescence intensity in arbitrary units.

It is important to consider that in eukaryotes, endogenous aminoacyl tRNA synthetases and their cognate tRNAs can be shuttled between the nucleus and the cytoplasm through the action of many different cellular processes and responses.^8^ However, the expression of an orthogonal pair (the GCE machinery) is likely to result in impeded GCE efficiency if subjected to similar processes. Whereas NLSs can occasionally be identified in prokaryotes (which include bacteria and archaea), their role in such organisms, which lack a nucleus, is widely debated.^9^ For the purpose of achieving efficient, high‐yielding GCE in general, we assume that it is neither desired nor expected for the tRNA/RS pair to be mainly localized to the nucleus and thus spatially separated from the translational machinery present in the cytoplasm.

To reinforce cytoplasmic localization, we added a strong nuclear export signal (NES)^10^ to the N terminus of the PylRS^AF^ (NESPylRS^AF^), which we hypothesized would outcompete any NLS import signal intrinsic to the PylRS. Indeed, IF and FISH staining revealed a clear cytosolic distribution of both the NESPylRS^AF^ and tRNA^Pyl^ (Figure 1 b,d and Figure S3). To test whether this also increases the efficiency of the system, we used fluorescence‐based flow cytometry of cells expressing an Amber suppression reporter (iRFP–GFP^Y39TAG^; iRFP is a near‐infrared fluorescent protein) in the presence and absence of an unreactive *tert*‐butoxycarbonyl lysine derivative (BOC) as the ncAA. Our reporter is composed of iRFP, which is fused to the Amber mutant of GFP (Y39TAG) at its C terminus. In this assay, full‐length iRFP–GFP is only produced if the TAG codon is suppressed to encode the ncAA. The intensity of the green fluorescence (GFP) indicates the efficiency of Amber suppression, while iRFP fluorescence reports whether the cells were properly transfected. As shown in Figure 1 e (and in detail in Figure S4), we observed an up to 15‐fold enhancement of Amber suppression efficiency with NESPylRS^AF^.

We next wanted to test whether this NESPylRS^AF^ construct also reduces background in fluorescence labeling experiments, in particular the unwanted nuclear background staining. We performed a side‐by‐side comparison of intracellular labeling experiments with tRNA^Pyl^/NESPylRS^AF^ and the conventional tRNA^Pyl^/PylRS^AF^ system. We used the axial atropisomer {[(*E*)‐cyclooct‐2‐en‐1‐yl]oxy}carbonyl)‐l‐lysine (TCO^*a^), which we previously determined to be an ideal choice for site‐specific labeling with 1,2,4,5‐tetrazine containing Cy5 dye (Cy5‐tet) derivatives,5a, 11 despite the possibility that a click‐reaction side product is also formed that could eliminate the dye from the protein.^12^ Figure 2 a,b shows Amber suppression results using TCO^*a^ of the transcription factor jun‐B^348TAG^–GFP labeled with Cy5‐tet using SPIEDAC (jun‐B^348→TCO*a→Cy5^). This construct contains a C‐terminal GFP fusion, which is only generated when the Amber codon is suppressed. In such a case, the jun‐B–GFP signal can be used as a reference to validate proper labeling.5b The detrimental effect of using the conventional system is particularly evident in Figure 2, where the Cy5 fluorescence (which should indicate jun‐B staining) shows a similar shape to a nucleolus. When inspecting only the Cy5 channel using the conventional system, nucleoli and areas of jun‐B^348→TCO*a→Cy5^ localization might be easily confused (Figure 2 a). This background signal can even be observed when no POI is introduced (Figure S5), and thus most likely originates from ncAA bound or coupled to tRNA and/or RS accumulating in the nucleus. However, for the NES system (Figure 2 b), the labeled protein and GFP signals co‐localize (see Figure S6 for a quantitative co‐localization analysis), and thus faithful identification of jun‐B through the use of GCE and click chemistry becomes possible. Besides enhanced expression efficiency, removal of the unwanted nucleolar background is thus beneficial for general fluorescence microscopy, ranging from “simple” confocal imaging (as shown in Figure 2 and Figure S5) to super‐resolved microscopy techniques like STORM/GSDIM/STED (analogously to Figure 2, see Figure S7 for SRM images of jun‐B^348→TCO*a→Cy5^).

**Figure 2:**
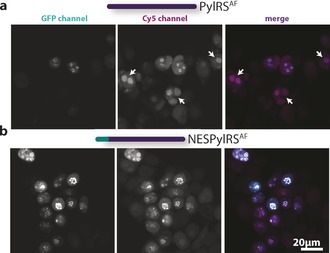
Confocal images of Cy5‐tet labeled jun‐B^348TAG→TCO*a^‐GFP expressed in HEK cells containing either tRNA^Pyl^/PylRS^AF^ (a) or tRNA^Pyl^/NESPylRS^AF^ (b). Left panel: GFP, central panel: Cy5, right panel: merge. Note the prevalent co‐localization of GFP and Cy5 in the NESPylRS^AF^ sample, contrary to the appearance of additional nonspecific nucleolar signal in PylRS^AF^ expressing cells (highlighted by arrows).

To extend the repertoire of GCE‐compatible SRM techniques, we next wished to demonstrate that our tRNA^Pyl^/NESPylRS^AF^ GCE‐based click labeling can be combined with the more recently developed DNA‐PAINT SRM^13^ in a “Click‐PAINT” approach. DNA‐PAINT relies on placing a short single‐stranded (ss) DNA (the “docking strand”) into the POI, to which a complementary ssDNA carrying a small photostable synthetic dye (the “imaging strand”) can transiently and reversibly anneal. By means of localization microscopy, the freely diffusing imaging strand can then be discerned from the annealed one and a super‐resolved image can be reconstructed.13b Although limited to fixed specimens, as the majority of SRM applications still are, the strength of DNA‐PAINT arises from several features, which will be summarized below. A particularly evident benefit for the combination of GCE with DNA‐PAINT is that the solubility and biocompatibility of many organic fluorophores can be enhanced by coupling them to a biomolecule like DNA, which leads to reduced tendency towards nonspecific binding (stickiness). In addition, GCE permits the imaging strand to be placed in direct proximity to the residue‐specifically installed ncAA. This is in contrast to previously described DNA‐PAINT, which was typically based on Ab labeling.^13^ Such approach introduces an Ab linker of up to 10 nm and can limit the accuracy of the method and lower the achievable labeling density, both of which can be crucial for optimal SRM (see Ref. 2, 14).

As outlined in Figure 3, first a DNA docking strand was equipped with a compatible 1,2,4,5‐tetrazine and reacted with the POI^TAG→TCO*a^. Next, an imaging strand containing the synthetic dye Atto655 was added to the cells. The dye was conjugated to the imaging strand such that upon annealing with the docking strand, it was in close proximity to the labeling site. To validate the method, we used a previously described Amber mutant of vimentin^N116TAG^.5b Figure 3 c shows an SRM image of our vimentin^N116→TCO*a→PAINT^–mOrange construct, which clearly gives enhanced resolution compared to the diffraction‐limited image from the mOrange reference channel (Figure 3 b).

**Figure 3:**
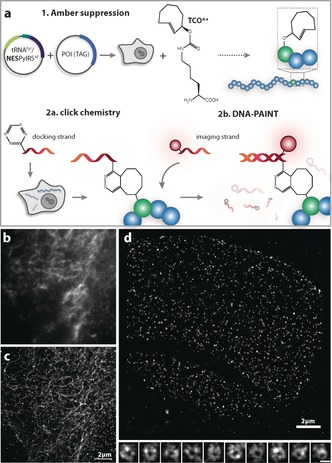
a) Schematic representation of the Click‐PAINT method. The POI^TAG^ is first expressed in mammalian cells in the presence of TCO^*a^ when co‐transfected with the tRNA^Pyl^/NESPylRS^AF^. Then, the POI^TAG→TCO*a^ is subjected to a two‐step labeling reaction in which first a tetrazine‐functionalized docking DNA strand is chemically ligated in a SPIEDAC reaction and second, a complementary imaging strand conjugated with a dye is added to the cells. b) Fluorescence signal of the fused mOrange protein for the vimentin^N116→TCO*a^–mOrange construct used as a reference for protein expression. c,d) DNA‐PAINT‐based SRM performed by acquisition in the channel that is appropriate for the dye introduced using the Click‐PAINT method for vimentin^N116→TCO*a→PAINT^–mOrange, resolution 50 nm (c), and GFP^N149→TCO*a→PAINT^–Nup153, resolution 25 nm (d; scale bar in insets with zoomed‐in nuclear pores is 100 nm, see also Figure S8). The resolution was determined using Fourier ring correlation.^2^

We next aimed to test whether the beneficial features of Click‐PAINT can enable more demanding microscopy studies involving the imaging of less abundant structures in cells, such as the nuclear pore complex (NPC), a ring‐like structure in the nuclear envelope. Thirty‐two copies of the protein nucleoporin Nup153 have been recently counted in the NPC,^15^ which has an approximate volume of 60 nm^3^. Labeling sites for Nup153 are consequently at a substantially lower abundance and density than those for cytoskeletal filaments. We generated a GFP^N149TAG^–Nup153 construct (with GFP serving as reference) and subjected it to our method. As shown in Figure 3 d, we were able to obtain super‐resolved images showing the typical circular appearance of NPCs by using total internal reflection fluorescence (TIRF) microscopy, which shows NPCs facing the coverslip and objective.^15^, ^16^ We note that not all rings are closed, since the cells also express wild‐type (unlabeled) Nup153, which will compete for incorporation into the NPC with our GFP^N149→TCO*a^–Nup153 protein.

In summary, we have eliminated a major flaw in the eukaryotic application of the most popular GCE system, the tRNA^Pyl^/PylRS pair from *Methanosarcina*. Undoubtedly, yield is still one of the major issues of GCE in general, and not just for SRM applications. The main reason for low yields is competition between the host's internal translation termination machinery and the stop codon suppression system. To address this issue in eukaryotes, many approaches, including promoter engineering, better evolution of the RS, and release factor engineering, to name just a few, have been developed. Increasing the amount of tRNA, for example through gene multi‐chaining, has been a major focus of many previous studies for GCE yield enhancement (for Reviews, see Refs. 3), simply because the lower the concentration of properly charged suppressor tRNA^Pyl^, the more likely it is that the eukaryotic release factor terminates translation. We discovered that PylRS contains an NLS and accumulates together with its cognate tRNA^Pyl^ in the nucleus, and that appending a NES to PylRS relocates the pair back to the cytoplasm, where it can be translationally active. Our repair (“debugging”) strategy is extremely easy to implement, since existing systems only require N‐terminal fusion of the NES to the PylRS. Therefore, every user of the eukaryotic pyrrolysine GCE machinery could immediately reap the benefits of enhanced codon suppression efficiency and thus a higher expression yield for any application that requires a more efficient system.

Cytoplasmic relocalization of the PylRS also results in increased contrast in labeling experiments, as most easily recognized by lack of nonspecific nucleolar staining (Figure 2). This enables contrast‐enhanced imaging of proteins within the nucleus, a compartment previously not faithfully accessible for GCE‐based labeling experiments. This is of benefit for all fluorescent‐dye‐based imaging modalities (from confocal microscopy to SRM techniques; Figure 2 and Figure S7).

In addition, we presented a combination of the enhanced GCE system with DNA‐PAINT, which we term Click‐PAINT, in an application to image even low‐abundance proteins in the nucleus. DNA‐PAINT has multiple features that make it a particularly powerful SRM technique in biology.^13^ For example, a large reservoir of imaging strands can help to reduce bleaching problems, and the technique has the potential to enable direct quantification of the number of fluorescent labels in an image. The latter is an important parameter with respect to the ultimate goal of direct quantification of protein concentration in cells through microscopy.13a

Another indirect benefit is that conjugating dyes to ssDNA makes many fluorescent probes biocompatible and soluble. This can increase the robustness and generality of the method in combination with GCE by lowering nonspecific dye staining, thus providing an alternative to the need for using fluorogenic dyes or optimization of the washing conditions, as is sometimes necessary when coupling dyes directly to ncAAs.^5^

We note that GCE still has many limitations with respect to SRM, such as the generation of truncated proteins and the suppression of natural Amber codons. These hurdles need to be addressed in the future, in particular when aiming to quantify the number of expressed proteins and not just fluorescence labels. However, while GCE‐based SRM is not yet as simple to implement as fluorescent protein fusions or antibody‐based labeling techniques, these techniques do not offer residue‐level precision and the versatility of placing a labeling site virtually anywhere in a protein.

Nevertheless, the combination of residue‐specific resolution of click chemistry‐based GCE with DNA‐PAINT clears the way for in‐cell structural biology experiments.

## Supporting information

As a service to our authors and readers, this journal provides supporting information supplied by the authors. Such materials are peer reviewed and may be re‐organized for online delivery, but are not copy‐edited or typeset. Technical support issues arising from supporting information (other than missing files) should be addressed to the authors.

Supplementary
