# Supplementary material for: Debugging Eukaryotic Genetic Code Expansion for Site‐Specific Click‐PAINT Super‐Resolution Microscopy
Source: Angew Chem Int Ed Engl. 2016 Nov 2;55(52):16172–6. doi: 10.1002/anie.201608284 (PMC5215487; doi:10.1002/anie.201608284)
Supplement: Supplementary file 1 — Supplementary [file ANIE-55-16172-s001.pdf]

Angewandte Chemie
Eine Zeitschrift der Gesellschaft Deutscher Chemiker

Supporting Information

Debugging Eukaryotic Genetic Code Expansion for Site-Specific Click-PAINT Super-Resolution Microscopy

*Ivana Nikić+, Gemma Estrada Girona+, Jun Hee Kang+, Giulia Paci, Sofya Mikhaleva, Christine Koehler, Nataliia V. Shymanska, Camilla Ventura Santos, Daniel Spitz, and Edward A. Lemke**

anie_201608284_sm_miscellaneous_information.pdf

Table of content Supplementary Information

Supplementary Methods

Cell culture

Constructs, cloning and mutagenesis

Transfections and ncAAs

Flow cytometry

PylRS immunolabeling, tRNA FISH and imaging

PylRS and jun-B labeling and imaging

Click-PAINT labeling

Click-PAINT and localization-based SRM

Super-resolution image processing

Supplementary Figures

Supplementary Figure 1: Prediction algorithms identify putative nuclear localization signals in *M. mazei* $PylRS^{AF}$ sequence

Supplementary Figure 2: Western blot of $PylRS^{AF}$ expressed in *E. coli*

Supplementary Figure 3: Cellular localization of $PylRS^{AF}$, $NESPylRS^{AF}$, and $tRNA^{Pyl}$ in COS-7 cells

Supplementary Figure 4: Amber suppression efficiency comparison in a reporter titration of $PylRS^{AF}$ vs $NESPylRS^{AF}$

Supplementary Figure 5: Background fluorescence labeling comparison of $PylRS^{AF}$ vs $NESPylRS^{AF}$

Supplementary Figure 6: Quantitative co-localization analysis of jun-B labeling

Supplementary Figure 7: Comparison of $PylRS^{AF}$ vs $NESPylRS^{AF}$ for super-resolution imaging of jun-B

Supplementary Figure 8: Detailed localization of individual nuclear pores

Supplementary Methods

Cell culture

HEK293T cells (ATCC CRL-3216) and COS-7 cells (Sigma 87021302) were maintained in Dulbecco's modified Eagle's medium (Life Technologies 41965-039) supplemented with 1% penicillin-streptomycin (Sigma P0781), 1% L-Glutamine (Sigma G7513), 1% sodium pyruvate (Life Technologies 11360), and 10% FBS (Sigma F7524). Cells were cultured at 37°C in a 5% CO_2 atmosphere and passaged every 2–3 days up to 15–20 passages.

In all cases, cells were seeded 15-20h prior to transfection at a density resulting in 70-80% confluency at the time of transfection. Poly-L-lysine (Sigma P5899) was used to coat the chambers for HEK293T experiments as described in ref. [1]. Immunolabeling and FISH were performed on 24-well plates with glass bottom (Greiner Bio-One). Four-well chambered Lab-Tek #1.0 borosilicate coverglass (ThermoFisher) were used for labeling and Click-PAINT experiments.

Constructs, cloning and mutagenesis

Amber stop codons were introduced in the cDNA of the different constructs via PCR-based site-directed mutagenesis. JUNB cDNA was obtained from genomic DNA of HEK293T and cloned into a pCI vector. A C-terminal GFP fusion was created and a TAG codon was introduced in between, generating the pCI-jun-B^{348TAG}-GFP construct. For vimentin, mutagenesis was performed on a pVimentin–PSmOrange plasmid, which was a gift from Vladislav Verkhusha (Addgene plasmid #31922),[2] and the TAG was introduced at position N116, generating the pVimentin^{N116TAG}–PSmOrange construct. For Nup153, a pGFP-Nup153 plasmid was first constructed by cloning a codon-optimized Nup153 cDNA into a pEGFP backbone. Later, the TAG was introduced at position N149 of the GFP gene, generating the pGFP^{N149TAG}–Nup153 construct. For the expression of the Amber suppression system in mammalian cells, we used pcDNA3.1-tRNA^{Pyl}/PylRS^{AF} plasmid, which contains the tRNA under control of a hU6 promoter and the synthetase under a CMV promoter, and the newly described pcDNA3.1-tRNA^{Pyl}/NESPylRS^{AF} plasmid (same as the former one but with an appended NES sequence (NH_2-ACPVPLQLPPLERLTLD-COOH) to the N-terminal of the PylRS^{AF}).

Transfections and ncAAs

All transfections were performed with the JetPrime reagent (PeqLab) according to the manufacturer's recommendations.

For Amber suppression system experiments cells were transfected at a ratio of a 1:1 with a POI^{TAG} vector and the tRNA^{Pyl}/PylRS^{AF} or tRNA^{Pyl}/NESPylRS^{AF} vectors (unless otherwise stated). TCO*[a] was added immediately after transfections. Eight to ten hours after transfection, the medium was exchanged to a fresh one (HEK293T cells) or a fresh one containing ncAA

(COS-7 cells). COS-7 cells were incubated for one more day and approximately 30–36h after transfection, the medium was exchanged to a fresh one.

Stock and working solutions for all the used ncAAs were prepared as described in ref.[1]. Unless otherwise stated, final concentration was 250 µM.

TCO*a, {[(E)-cyclooct-2-en-1-yl]oxy}carbonyl)-L-lysine and **BOC**, *t*-butyloxycarbonyl-lysine, were purchased from SiChem and IBIS respectively.

Flow cytometry

Two days after transfection (unless otherwise stated), cells were harvested, resuspended in 1xPBS and passed through 70 µm cell strainers. Transfections for flow cytometry were performed with three plasmids (POI^{TAG}, $tRNA^{Pyl}$, and the wanted version of synthetase) at a 1:1:1 ratio with 1.2 µg total DNA. Medium was exchanged for fresh medium containing the ncAA 4-6h post-transfection and left until the time of harvesting. Data acquisition was performed in an LSRFortessa SORP Cell Analyzer (BD). Analysis was done using the FlowJo software (FlowJo). Cells were first gated by cell type (using FSC-A x SSC-A parameters) and then by single cell (FSC-A x SSC-W). Lastly, fluorescence was acquired in the 488-530/30 channel for GFP signal and in the 640-730/45 channel for iRFP signal.

PylRS immunolabeling, tRNA FISH and imaging

For immunolabeling experiments, cells were fixed (one day after transfection) in 2% paraformaldehyde in 1xPBS at room temperature (RT) for 10 minutes. Permeabilization was performed with 0.5% Triton solution in 1xPBS for 15 minutes at RT. Samples were later blocked in 3% BSA in 1xPBS for 90 minutes at RT, after which incubation with the primary antibody (Ab_{PylRS}) was done overnight at 4°C at a concentration of 1 µg/ml in blocking solution. The next day, secondary antibody (ThermoFisher A-11007) was used at 2 µg/ml in blocking solution for 60 minutes at RT. Lastly, DNA was stained with Hoechst 33342 (Sigma B2261) at 1 µg/ml in 1xPBS for 10 minutes at RT. Washes in between steps were done in 1xPBS.

Fluorescence *in situ* hybridization (FISH) experiments were performed one day after transfection. The hybridization protocol was adapted for 24-well plates from Ref. [3]. The hybridization probe (5'-(DIG)CTAACCCGGCTGAACGGATTTAGAGTCCATTCGATC-3') was used at 0.16 µM. After the SSC washes, cells were incubated for 1h at RT in blocking buffer (0.1 M TrisHCl, 150 mM NaCl, 1x blocking reagent (Sigma 000000011096176001). Then, cells were incubated with anti-Digoxigenin-Fluorescein antibody (Sigma 000000011207741910) at a 1:200 dilution in blocking buffer ON at 4°C. The next day, 3 washes of 5 minutes were done in Tween buffer (0.1 M TrisHCl, 150 mM NaCl, 0.5% Tween20). Finally, DNA staining was performed as described above.

Confocal images were acquired on a Leica SP8 STED 3X microscope using the 405 nm (for Hoechst), 488 nm (for fluorescein) and 594 nm (for Alexa594) laser lines for excitation. For HEK293T 63x/1.40 Oil and for COS-7 40x/1.10 water objectives were used.

PylRS and jun-B labeling and imaging

One day after transfection, HEK293T cells were fixed in 2% paraformaldehyde in 1xPBS at room temperature (RT) for 10 minutes. Permeabilization was performed with 0.1% Triton solution in 1xPBS for 15 minutes at RT. Fixed cells were incubated for 10 minutes at RT with 1.5 µM sulfonated Cy5-tetrazine (sulfo-Cy5 tet, Jena Bioscience, cat. no. CLK-015) in 1xPBS (500 µM stock solution in DMSO) for localization-based SRM, or for 10 minutes at 37°C with 5 µM for confocal imaging. Washes in between steps were done in 1xPBS. Confocal images were acquired on a Leica SP8 STED 3X microscope with a 40x/1.10 water objective using the 488 nm (for GFP channel) and 635 nm (for Cy5 channel) laser lines for excitation. For details on SRM imaging see below.

Click-PAINT labeling

Approximately 24h after transfection (HEK293T) or 48h after transfection (COS7), cells were fixed with 2% paraformaldehyde in 1xPBS at RT for 10 minutes. After fixation, cells were permeabilized with 0.1% Triton (in 1xPBS) for 15 minutes at RT and rinsed with PBS, prior to the labeling step. For Click-PAINT labeling, the docking strand oligo (5'-ttatacatcta-3'), functionalized with 1,2,4,5-tetrazine at 5' end was purchased from biomers.net. A final concentration of 15 µM in 1xPBS prepared from a stock (500 µM in distilled water) was used. Labeling was performed at 37 °C for 10 minutes. After labeling cells were rinsed with 1xPBS. Prior to imaging, the imaging strand (5'-ctagatgtat-3') functionalized with Atto655 at the 3' end (biomers.net) was added to the cells at a final concentration of 800 pM (in 1xPBS, 500 mM NaCl, pH 8, as described in ref. [4]).

Click-PAINT and localization-based SRM

After fixation and labeling, cells were taken to the microscope, either on the same day or a maximum of 1–3 days later. Click-PAINT SRM imaging of vimentin and Nup153, and localization-based SRM imaging of jun-B was performed on a commercial Leica GSD microscope, equipped with a Leica HCX PL APO 160x/1.43. Oil CORR TIRF PIFOC objective. Transfected cells were identified with 532 nm (mOrange fusion) and 488nm (GFP fusion) excitation lasers. Atto655 and Cy5 were excited with a 642nm laser and images were acquired in TIRF mode for Click-PAINT and HiLO[5] for localization-based SRM imaging. For each image, 30000–40000 frames were acquired with 100 ms exposure for Click-PAINT and 30 ms for localization-based SRM, for which we used an imaging buffer according to a published protocol.[6]

Super-resolution image processing

Super-resolution images were reconstructed using the Localizer Package [7] for IgorPro (Wavemetrics, Portland, OR). Firstly, a threshold based on the maximum likelihood ratio was applied, followed by fitting with a symmetrical 2D Gaussian function for localization of the spots. In Click-PAINT, sporadic long-lasting associations of docking and imaging strands were observed which led to repetitive localization in sequential frames. In order to correct for this, identical emitters (falling within one standard deviation of the spot fit) were consolidated into a single intensity-weighed localization. For both cases, a final super-resolution image was reconstructed from binning all the detected events and convolving the resulting image with a Gaussian width according to the resolution determined by the Fourier ring correlation 2σ criterion for Nup153 and jun-B and 0.143 criterion for vimentin.[8]

Supplementary Figures

Supplementary Figure 1: Prediction algorithms identify putative nuclear localization signals in *M. mazei* PylRS^{AF} sequence

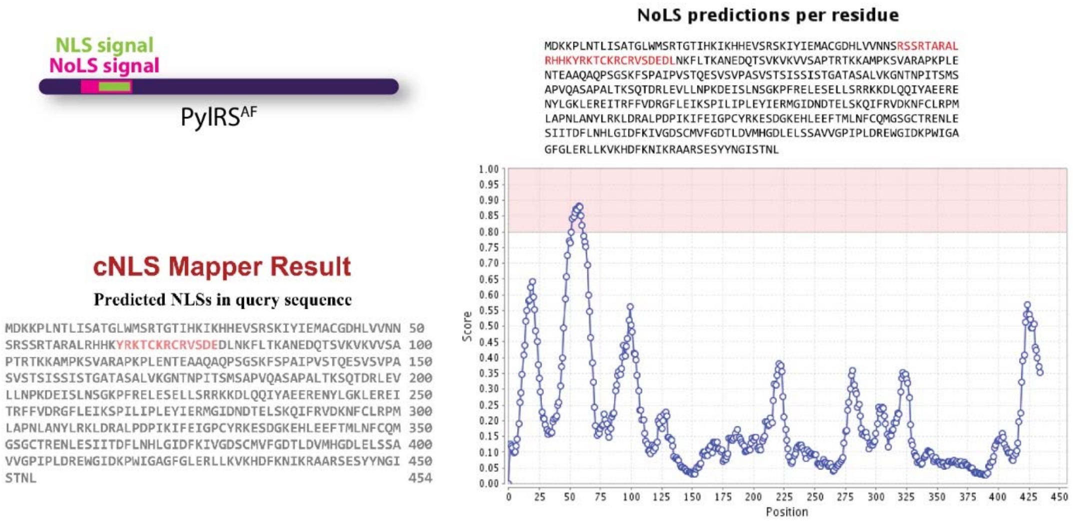

Computational analysis of the amino acid sequence of the PylRS^{AF} from *M. mazei* predicted putative nuclear as well as nucleolar localization signals (NLS and NoLS respectively). NLS and NoLS motifs prediction was done by using "cNLS Mapper" [9] and "NoD: NucleOlar localization sequence Detector".[10]

We note that for *M. barkeri* an NLS can be predicted as well.

Supplementary Figure 2: Western blot of PylRS^{AF} expressed in *E. coli*

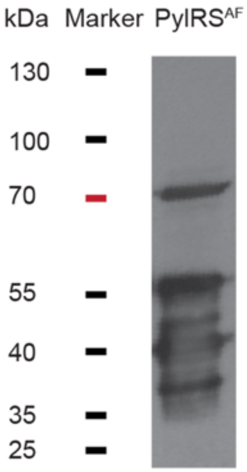

The expression of *M. mazei* PylRS^{AF} in Bl21(DE3)AI *E. coli* cells could be shown by Western blot (band at 55 kDa), using anti-PylRS (Ab_{PylRS}) as primary (Eurogentec) and anti-rat-HRP conjugate (Dianova) as secondary antibody. The marker lane is added manually after blotting.

Expression and purification of 6His-TEV-PylRS^{AF} for antibody production

The plasmid pTXB3-6His-TEV-PylRS^{AF} was transformed into Bl21(DE3)AI cells and 6His-TEV-PylRS^{AF} was expressed in TB-FB medium overnight at 18°C after induction with 0.02% arabinose and 1 mM IPTG. After harvesting the expression, the cell pellets were resuspended in 4xPBS (pH 8, 1 mM PMSF, 0.2 mM TCEP) and lyzed using a microfluidizer. The clear supernatant was obtained by spinning down the lysate for 1 hour at 15000 rpm at 4°C and incubated on Nickel beads for 1 hour at 4°C. After washing the Nickel beads with increasing imidazole concentrations, 6His-TEV-PylRS^{AF} was eluted from the Nickel beads using 400 mM imidazole in 4xPBS buffer. This elution fraction was concentrated using a protein filter device (Spin-X UF, Corning, 30 kDa cutoff) and loaded on a Superdex 200 column (GE Healthcare). The peak fractions were concentrated and used for immunization of two rats (Eurogentec). The resulting polyclonal anti-PylRS antibody was used for Western blot in a 1:400 dilution. As a secondary antibody, anti-rat (Dianova) was applied in a 1:5000 dilution. The chemiluminescent signal was obtained with an ECL Kit (GE Healthcare).

Supplementary Figure 3: Cellular localization of $PylRS^{AF}$, $NESPylRS^{AF}$, and $tRNA^{Pyl}$ in COS-7 cells

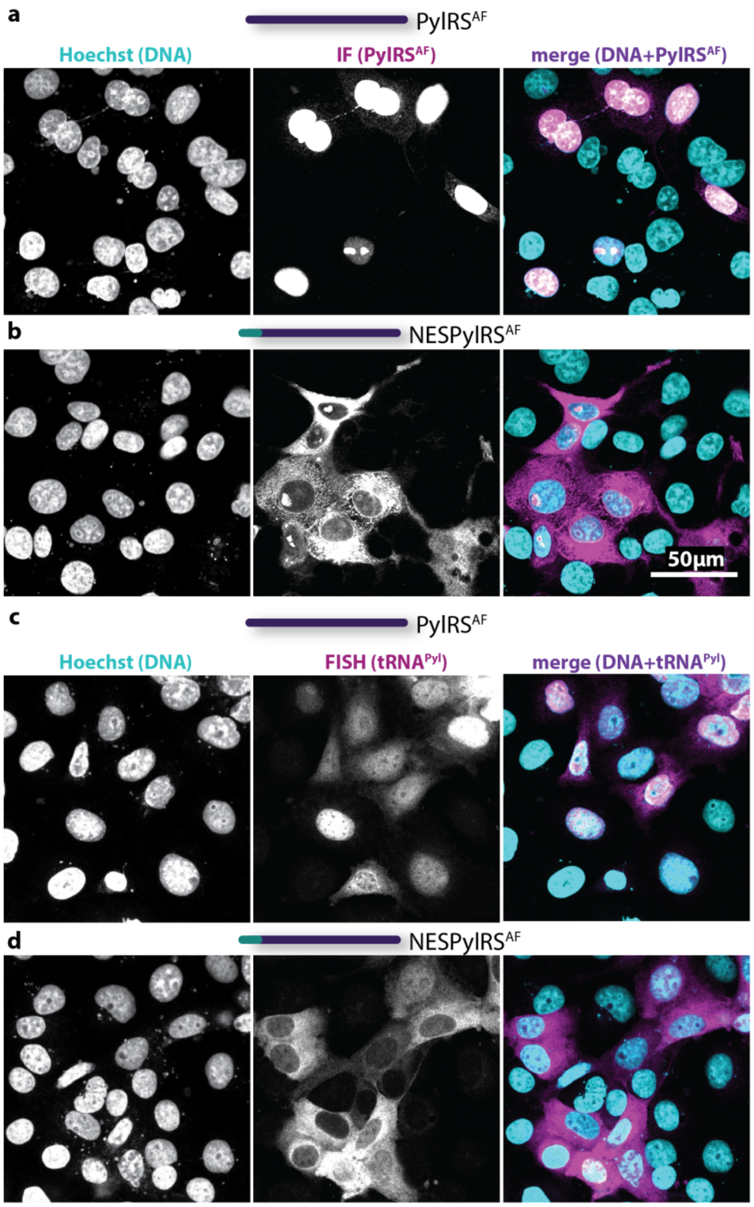

Analog to the images of HEK293T cells in **Figure 1**, here we show the immunofluorescence of $PylRS^{AF}$ and the FISH for $tRNA^{Pyl}$ in COS-7 cells containing either the $tRNA^{Pyl}/PylRS^{AF}$ (a,c) or $tRNA^{Pyl}/NESPylRS^{AF}$ (b,d). Left panel: Hoechst 33342, central panel: Ab_{PylRS} staining (for a,b) and FISH $tRNA^{Pyl}$ (for c,d), right panel: merge. In (a) a strong nuclear and putatively nucleolar signal is clearly observed, whereas almost no signal is detected in the cytoplasm. Contrarily, in (b) the synthetase is mainly localized in the cytoplasm. Re-localization of the synthetase results in a simultaneous re-distribution of the tRNA signal mostly in the cytoplasm (d), in contrast to a much more heterogeneous and with strong nuclear signal for the conventional system (c).

Supplementary Figure 4: Amber suppression efficiency comparison in a reporter titration of PylRS^{AF} vs NESPylRS^{AF}

Amber suppression efficiency in eukaryotic cells is dependent on various factors, such as ncAA concentration and level of reporter expression. To account for this we show two full reporter titration experiments for comparison of the tRNA^{Pyl}/PylRS^{AF} and the tRNA^{Pyl}/NESPylRS^{AF} systems. HEK293T cells were co-transfected with one of those pairs and a varying amount of the iRFP-GFP^{Y39TAG} Amber suppression reporter, ranging from 100 ng to 500 ng of plasmid DNA in steps of 100 ng (top to bottom row in flow cytometry subfigures below). Furthermore, we performed the experiment both with a low concentration of ncAA (50 µM BOC, "low concentration ncAA" subfigure) and a high concentration (250 µM BOC, "high concentration ncAA" subfigure), representing conditions for low and high Amber suppression efficiency. Data was acquired 16 hours post-transfection. For each combination a sample without ncAA is also shown. The raw data from the "low concentration ncAA" subfigure was summed up across all DNA concentrations to generate the average plot shown in **Figure 1e.**

We provide two complementary analysis to asses efficiency and increased potential for imaging of the system.

i) We first aimed to compare the efficiency of the new versus the conventional system by looking at the percentage of cells that become positive when tested under the same conditions. For that, we retrieved the total number of events on the double positive cells gate (DPs, named iRFP,GFP in flow cytometry plots) and normalized them against the total number of transfected cells (i.e. iRFP+iRFP,GFP gates) for each of the tested conditions. The results, shown in the table below, clearly summarize that the use of the NESPylRS^{AF} greatly enhances the suppression yield.

ncAA	ng of reporter DNA	system	total DPs events	% DPs	system	total DPs events	% DPs
low concentration	100	NES PylRS^{AF}	2981	**22.8**	PylRS^{AF}	376	**2.3**
	200		4550	**23.5**		766	**4.7**
	300		5218	**24.4**		987	**5.1**
	400		4616	**20.5**		1066	**5.0**
	500		3851	**20.0**		928	**4.1**
high concentration	100	NES PylRS^{AF}	7972	**30.7**	PylRS^{AF}	3962	**18.0**
	200		8665	**31.9**		5444	**21.4**
	300		9217	**33.8**		6618	**27.9**
	400		10607	**37.9**		7092	**26.3**
	500		9485	**34.6**		6159	**26.7**

ii) Additionally, we set to pay special attention on whether not only the amount but the intensity of positive cells changes when the use of the NESPylRS^{AF} is implemented. The analysis of the fluorescence intensity efficiency change is of particular interest especially in applications such as SRM where the user needs to extensively search for bright cells to image on a large and very heterogeneous population (besides biological variation of expression, multi-plasmid transfection does contribute to a heterogeneous cell population) of cells.

For that we set three gates within the DPs gate and classified the events within as dim, bright and very bright DPs (see top left panel in the flow cytometry plots below for a visualization of these gates). The setting of these gates was based on recurrent patterns that we have extensively observed when testing systems with varying efficiency. From this classification, we then calculated the fold change of the number of cells for NESPylRS^{AF} for each gate relative to PylRS^{AF} under the same conditions. This data is summarized in the bar plots below.

The bar plot provides an analysis of the fold change of the number of cells within each gate for NESPylRS^{AF} vs PylRS^{AF} across all tested conditions (5 different reporter concentrations under two different concentrations of ncAA). The bar plots were generated from the flow cytometry shown below (flow cytometry data for the low concentration of BOC (50 µM), and for high concentration (250 µM)).

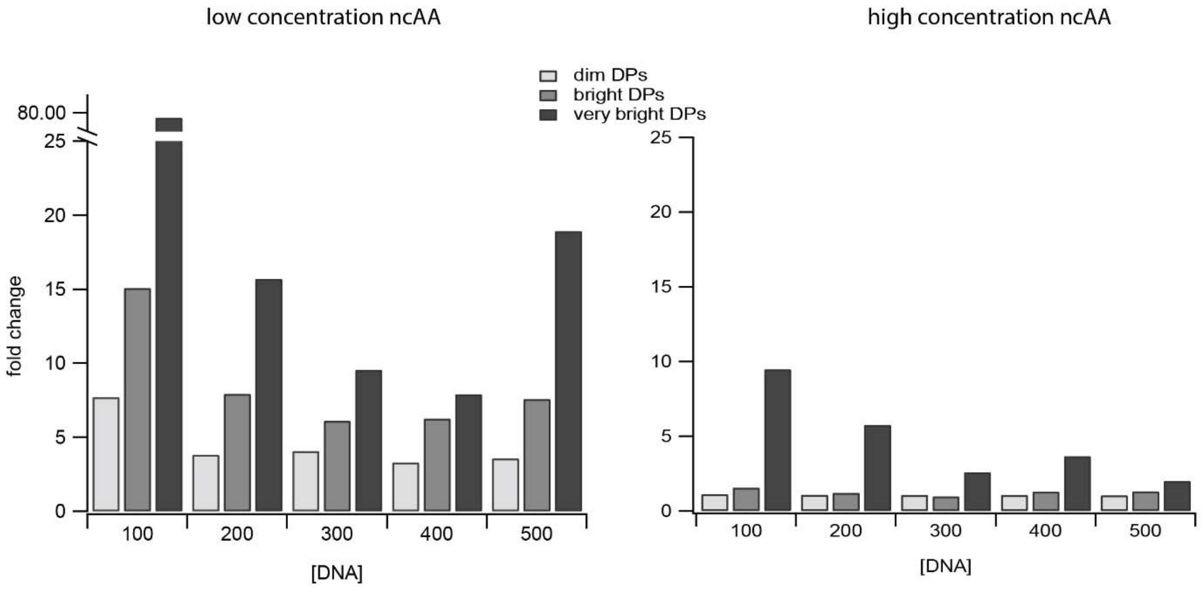

Fold changes were calculated as follows: first we normalized the total amount of events acquired in each of the gates by the total amount of events acquired and classified as transfected (e.g. #dim cells / #total iRFP,GFP + iRFP cells). Then, in order to calculate the difference in efficiency between +/-NES samples, each (+)NES condition (DNA amount and BOC concentration) was normalized against its (-)NES counterpart (e.g. #dim cells (normalized) in (+)NES-lowBOC-100ng / #dim cells (normalized) in (-)NES-lowBOC-100ng). The result of this division represents the percentage of improvement when using the NES system. These percentages are shown in the barplots, where they are plotted in fold change (i.e. 1500% becomes 15-fold). For each of the tested conditions the fold enhancement does vary but the improvement of the NESPylRS system is consistent and in particular the number of bright cells (the ones that in an imaging experiment are typically selected for) is increased dramatically (up to ~15 fold).

For high concentration of BOC the fold changes are in average lower, as the cells are near saturation conditions of producing reporter protein.

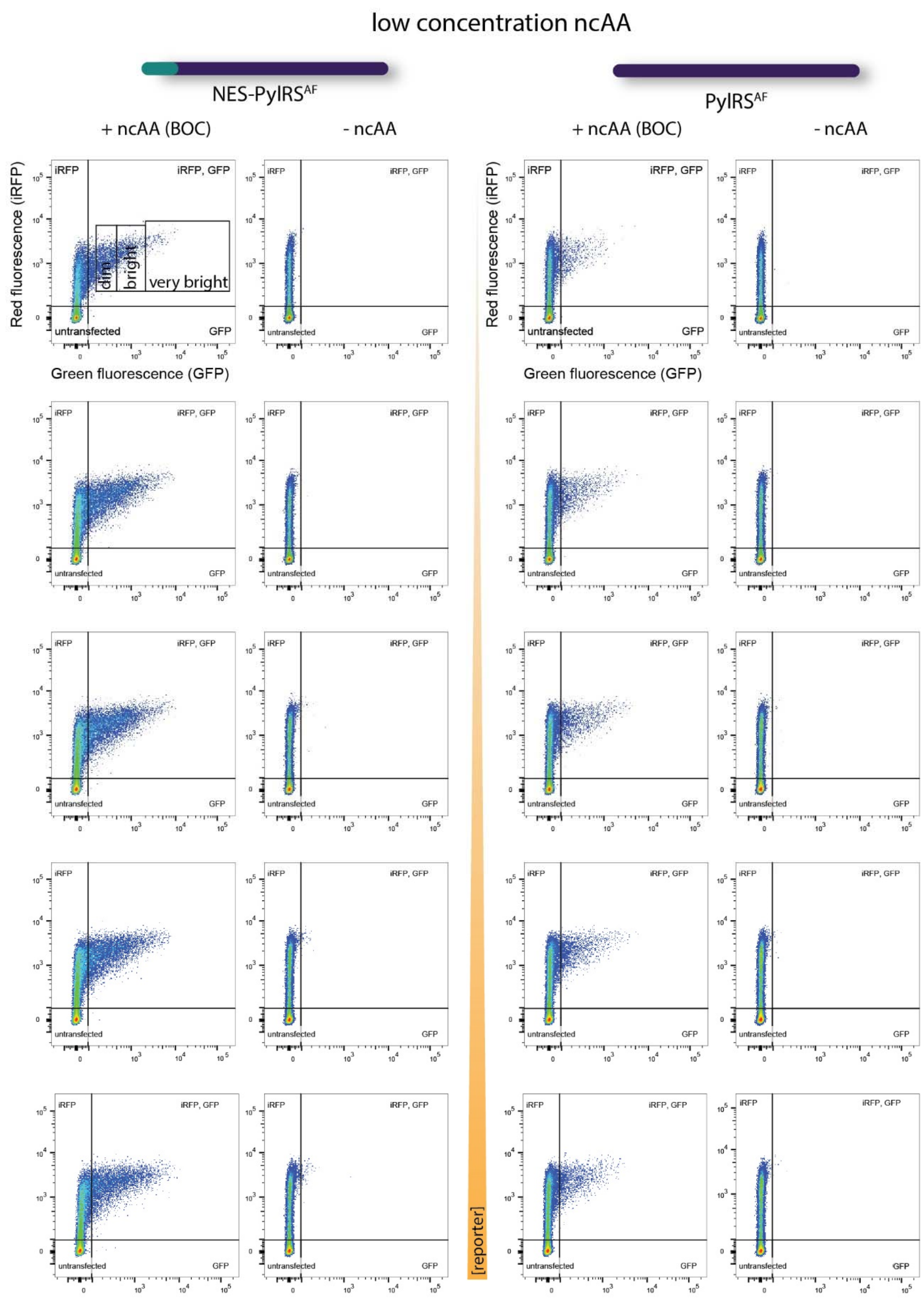

low concentration ncAA
NES-PylRS^AF
PylRS^AF
+ ncAA (BOC)
- ncAA
Red fluorescence (iRFP)
Green fluorescence (GFP)
iRFP
iRFP, GFP
untransfected
GFP
dim
bright
very bright
[reporter]

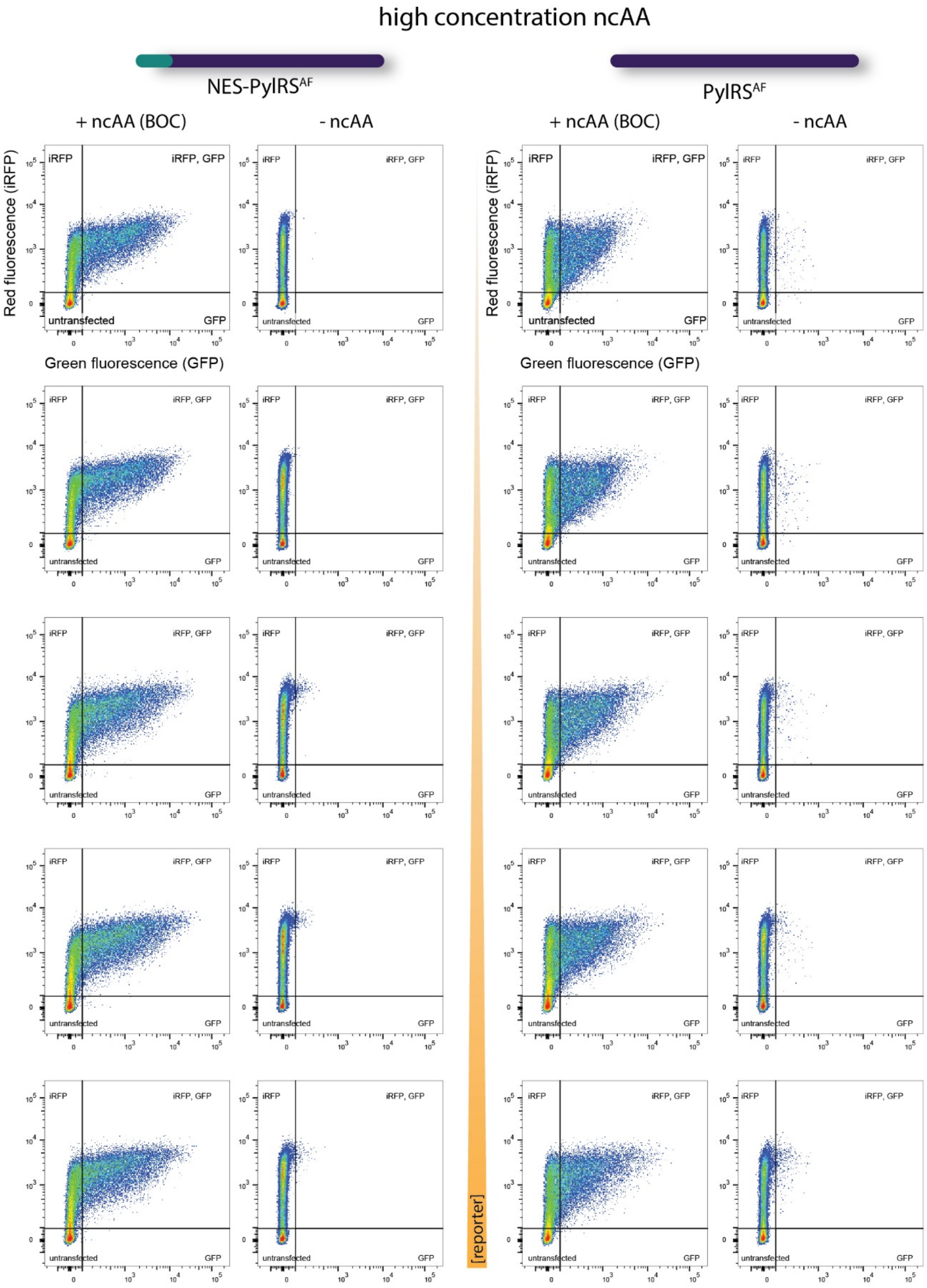
high concentration ncAA
NES-PylRS^{AF}
PylRS^{AF}
+ ncAA (BOC)
- ncAA
Red fluorescence (iRFP)
Green fluorescence (GFP)
iRFP
iRFP, GFP
untransfected
GFP
[reporter]

Supplementary Figure 5: Background fluorescence labeling comparison of $PylRS^{AF}$ vs $NESPylRS^{AF}$

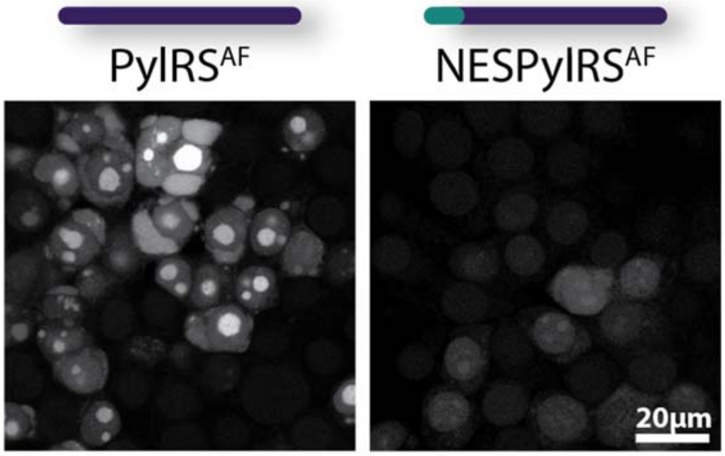

Confocal images of Cy5-tet labeled HEK293T cells expressing either $tRNA^{Pyl}$/$PylRS^{AF}$ (left) or $tRNA^{Pyl}$/$NESPylRS^{AF}$ (right).

Supplementary Figure 6: Quantitative co-localization analysis of jun-B labeling

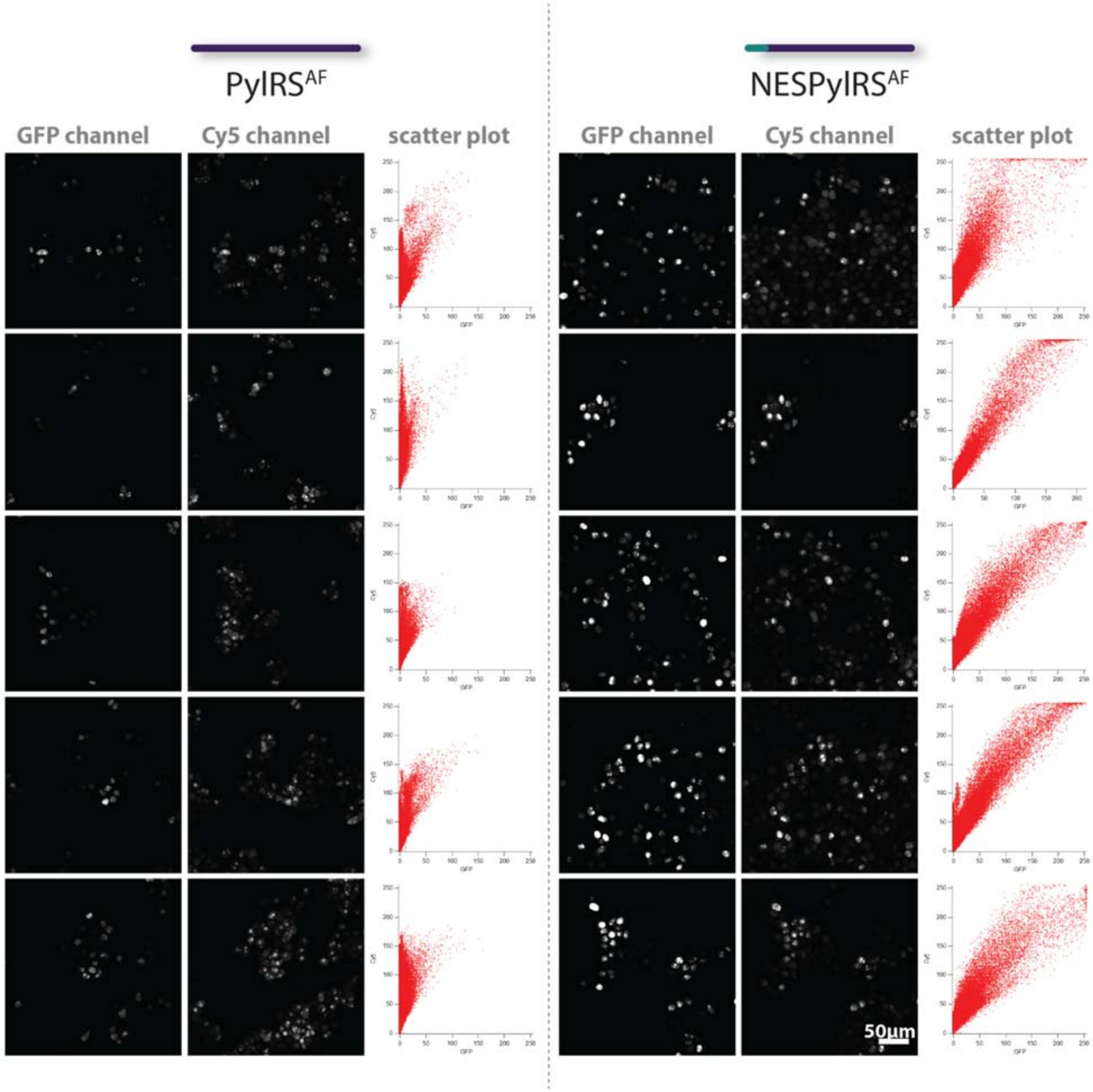

Quantitative comparison of nonspecific nucleolar labeling in HEK cells expressing jun-B$^{348TAG\rightarrow TCO*a}$-GFP when using the conventional versus the new system. Left panel: GFP (jun-B expression), central panel: Cy5 (labeling), right panel: scatter plot. The scatter plot displays the intensity of a given pixel in the GFP and the Cy5 channel (x- and y-coordinate respectively). A diagonal trend is observed for NESPylRS^{AF} which signifies co-localization between the two channels. On the other hand, for PylRS^{AF}, scattering is observed at the y-axis due to presence of nonspecific nucleolar signal which does not co-localize in the GFP channel. All the shown images were quantitatively analyzed using ImageJ to give Mander's coefficient which describes the proportion of the signal in the Cy5 channel co-localizing with the GFP channel. [11] The

average value of Mander's coefficient for PylRS^{AF} is 0.19 while for NESPylRS^{AF} is 0.85 and thus substantially higher.

Overlap coefficients corresponding to above images given in order (top to bottom) for PylRS^{AF}: 0.265, 0.087, 0.229, 0.230, 0.128

Analogously, overlap coefficients in order (top to bottom) for NESPylRS^{AF}: 0.649, 0.958, 0.856, 0.877, 0.932.

Supplementary Figure 7: Comparison of PylRS^{AF} vs NESPylRS^{AF} for super-resolution imaging of jun-B

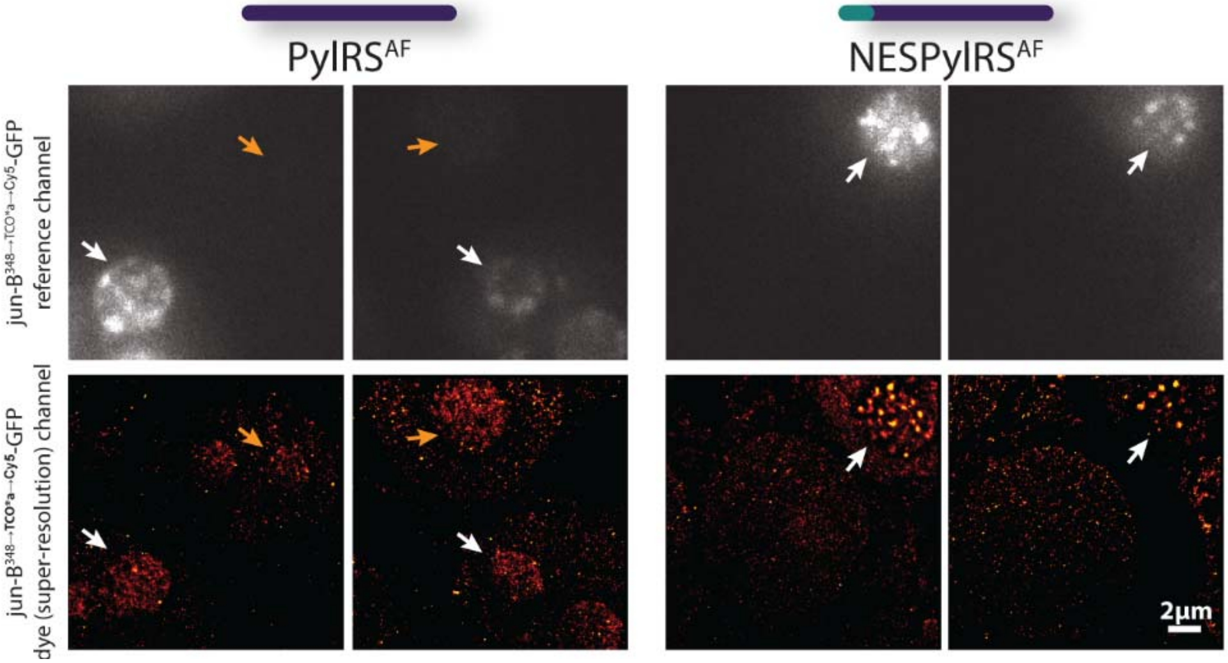

Analogously to the confocal images of jun-B$^{348TAG\rightarrow TCO*a\rightarrow Cy5}$-GFP in **Figure 2** maintext, we show here super-resolution images obtained using ground state depletion followed by individual molecule return (GSDIM) (bottom row panels). For reference, the top row panels show the corresponding GFP fluorescence signal. Notice that for tRNA^{Pyl}/PylRS^{AF} (left), in the GSDIM channel staining appears (orange arrow), which could be easily confused for jun-B$^{348TAG\rightarrow TCO*a\rightarrow Cy5}$ (white arrows), but that lack a corresponding GFP reference signal from jun-B expression in the GFP channel. For the NESPylRS^{AF} system, the co-localization is always apparent, and the signal to noise also appears better.

Images were acquired in the equatorial plane of the cell using HiLo mode. [5] No 3D SRM technique was applied that could further enhance overall image quality. The resolution of the images (left to right) is: 37, 24, 25, and 29 nm and was determined using Fourier ring correlation (FRC). [8]

Supplementary Figure 8: Detailed localization of individual nuclear pores

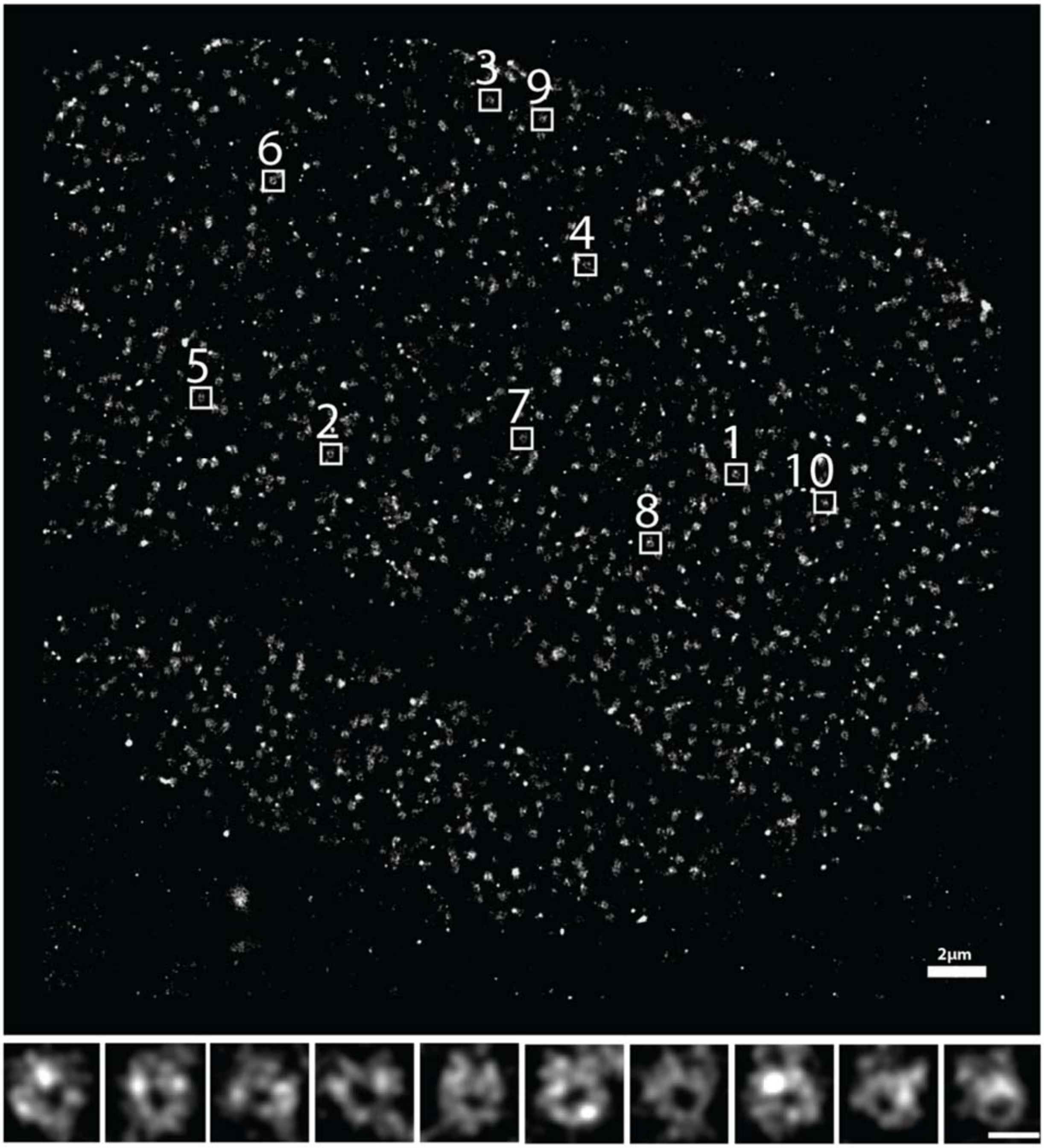

Super-resolution image of $GFP^{N149 \rightarrow TCO^{*}a \rightarrow PAINT}$–Nup153 construct labeled via the Click-PAINT protocol. The image is the same as the one shown in the main text **Figure 3d**. Here we highlight the specific regions that were chosen on the whole cell footprint to generate the 10 zoomed-in nuclear pores complexes depicted on the bottom. The numbering follows the order of the position of the zoomed-in images from left to right (1 to 10).

Supplementary references

[1] I. Nikic, J. H. Kang, G. E. Girona, I. V. Aramburu, E. A. Lemke, *Nature protocols* **2015**, *10*, 780-791.

[2] O. M. Subach, G. H. Patterson, L. M. Ting, Y. Wang, J. S. Condeelis, V. V. Verkhusha, *Nature methods* **2011**, *8*, 771-777.

[3] J. B. Pierce, S. C. Chafe, M. B. Eswara, G. van der Merwe, D. Mangroo, *Methods in cell biology* **2014**, *122*, 415-436.

[4] R. Jungmann, M. S. Avendano, J. B. Woehrstein, M. Dai, W. M. Shih, P. Yin, *Nature methods* **2014**, *11*, 313-318.

[5] M. Tokunaga, N. Imamoto, K. Sakata-Sogawa, *Nature methods* **2008**, *5*, 159-161.

[6] G. T. Dempsey, J. C. Vaughan, K. H. Chen, M. Bates, X. Zhuang, *Nature methods* **2011**, *8*, 1027-1036.

[7] P. Dedecker, S. Duwe, R. K. Neely, J. Zhang, *Journal of biomedical optics* **2012**, *17*, 126008.

[8] N. Banterle, K. H. Bui, E. A. Lemke, M. Beck, *J Struct Biol* **2013**, *183*, 363-367.

[9] S. Kosugi, M. Hasebe, M. Tomita, H. Yanagawa, *Proceedings of the National Academy of Sciences of the United States of America* **2009**, *106*, 10171-10176.

[10] M. S. Scott, P. V. Troshin, G. J. Barton, *BMC bioinformatics* **2011**, *12*, 317.

[11] S. Bolte, F. P. Cordelieres, *Journal of microscopy* **2006**, *224*, 213-232.
